# Miscellaneous

**Published:** 1864-10

**Authors:** 


*Antidotes for Strychnia.*—Professor R. Bellini, after conducting a long series of experiments on poisoning by strychnia and its salts, arrives at the opinion, that the best antidotes are tannic acid or tannin, chlorine, and the tinctures of iodine and bromine. Chlorine, he maintains, attacks the strychnia, even when it is diffused through the system, for he found that in rabbits poisoned with the sulphate of the alkaloid, on being made to inhale chlorine gas in quantity, such as was not sufficient in itself to kill, the convulsions were retarded, and were milder when they occurred; death was also less rapid. The author further observed, that when strychnia was exhibited with pyrogallic acid, convulsion was retarded for the space of half an hour, by comparison, with other experiments in which the alkaloid was given by itself. Professor Bellini believes that this arrest in symptoms is not dependent on acid acting chemically on the strychnia, but only through the astringent effects produced by the acid on the mucous membrane of the stomach whereby the absorption of the poison is rendered difficult. The same author, dwelling on the frog-test for strychnia, asserts that this test is not to be trusted, inasmuch as other poisons produce the tetanic symptoms, although in a lesser degree.—*Brit. Med. Jour.*

—The Royal Society for the Prevention of Cruelty to Animals, offers through the London *Times* a prize of fifty pounds for the best essay on the vivisection of animals, under the following propositions: 1st. Is it necessary to give dexterity to the operator? 2d. Is it necessary or justifiable for the general purposes of science, and if so, under what limitations?

*A Cause of Bronchitis.*—It has been found, in France, that the use of threshing machines has produced an immense amount of bronchitis and disease of the throat among the laborers employed, who are exposed to an atmosphere charged with dust, which affects them so powerfully, that in some parishes there are whole families of confirmed invalids. An order has been issued that the laborers employed near these machines must work in veils.

*The Tomb of Hippocrates.*—According to an Athenese journal, this tomb has been recently discovered near the village of Arnaoulti, not far from Pharsalia. An inscription leaves no doubt as to the identity of the original inhabitant of this sepulchral structure. In the interior were found a gold ring in the form of a serpent, the antique symbol of the curing art, a small chain and band of the same metal. A bust in bronze was also discovered, which is presumed to be a likeness of Hippocrates. These objects, together with the inscribed stone, were given, by the Turkish inhabitants of the district to Hourni Pasha, Governor of Thessalp, who forwarded them to Constantinople.

---

*An Association of American Ophthalmic Surgeons.*—In accordance with arrangements previously made, a convention of gentlemen devoted to Ophthalmological science and practice was held at the New York Eye Infirmary, during the late meeting of the American Medical Association. Dr. Delafield of New York, presided, and delegates were present from various parts of the United States. It was voted to hold the first annual meeting in the city of New York on the second Tuesday of June, 1865.

---

*Proportion of Births to Population.*—The proportion of births to population in various European countries, is given in a blue-book of "Statistical Tables relating to Foreign Countries." In England and Wales the annual births are 1 in 28 persons; 1 in 30 in Belgium, Holland and Norway; 1 in 32 in Sweden; 1 in 33 in Hanover, the Hans Towns, and Denmark; 1 in 34 in Greece; 1 in 38 in France; 1 in 26 in Wurtemburg; 1 in 25 in Russia; 1 in 24 in Austria, Saxony and Prussia; and 1 in 23 in Poland.

---

*Iodide of Potassium and Belladonna in Rheumatism.*—Dr. White, of Summit Hill, Penn., states in the *Med. Repor*-

*ter*, that he has used the iodide of potassium and tinc. belladonna with good results in acute rheumatism. He gives a teaspoonful of the following mixture every four hours, iodide potass., ℨ ij, tinct. belladonna, ℨ ij, aqua cinnamoni, ℥ ivss. This forms a very good alterative and anodyne, but the dose is too small.

---

*Ligature of the Femoral Artery for the Cure of Elephantiasis of the Leg and Foot.*—The N. C. *Med. Journal* says:—"Dr. J. L. Ozier reports (Charleston *Med. Journal*,) a case of elephantiasis of the leg and foot, of enormous size, which had existed for five years, in a negro man, 26 years of age, in which he applied a ligature to the femoral artery. At the time of the report, three months since the operation, it is stated that 'the leg and foot have subsided to very nearly their natural size.'"

---

*Discoverer of Anæsthesia.*—A pamphlet of fifteen pages is in circulation, setting forth the claims of Dr. Horace Wells, of Hartford, Conn., as the real discoverer of anæsthesia. The evidence, to say the least, is very much in his favor.

---

—Robert M. DeWitt, of New York, has in press, and will publish in a few weeks, a new work on "Morbid Tumors," by Rudolph Virchow, Professor of Pathological Anatomy, Berlin.

---

*Obituary.*—Died, at Pinkhill, near Edinburg, June 17th, 1864, aged 52, James Miller, M. D., for 22 years the able Professor of Surgery in the Unviersity of Edinburg, and author of a well known system of Surgery.

---

—The daughter of Priessnitz, the founder of cold water cure and a non-believer in vaccination, recently died in Kaschau ot small-pox.

—From a comparison of the statistics of 21 nations in Europe and America, it appears that one blind person is found among 1267 persons. The limits are: in America, 1 in 2489; in Norway, 1 in 540.

---

—Messrs. Sangman & Co. announce the publication of an abridgement of Copland's Medical Dictionary, brought down to the present state of Medical Science by the author. And Comparative Anatomy and Physiology of Vertebrate Animals, by Prof. Owen. Mr. R. B. Carter has translated Zander's "Ophthalmoscopi: its varieties and uses," from the German.

---

—The most recent excavations of Pompeii have yielded, besides a bronze statuette of Silenus, another most important discovery. Hitherto, no well with water had ever been found in Pompeii; during these excavations, however, a subterranean room was laid open, with a well 25 mitres in depth, in which the most excellent drinking water was found.

---

*The Negro in Disease.*—In the department of the South there are a number of regiments of colored troops, and it is a well ascertained fact that they are more liable to disease, and that the mortality is greater than among the white regiments. They rarely ever recover from a severe wound, and when attacked by disease they seem to care but little for life, and die in spite of all remedies and attention. These facts are particularly true of the North Carolina and South Carolina colored soldiers, the sick reports of which are fifty per cent. larger than those of the white troops; and I find, on referring to my notes, that there were, during the months of November and December, thirty-eight deaths from disease in thirteen regiments, three of which were colored. The latter lost seventeen men of the thirty-eight. The colored troops recruited in the Northern States do not suffer to the same degree.—*Dr. Goss, in American Medical Times.*

